# The Histone Methyltransferase Inhibitor A-366 Uncovers a Role for G9a/GLP in the Epigenetics of Leukemia

**DOI:** 10.1371/journal.pone.0131716

**Published:** 2015-07-06

**Authors:** William N. Pappano, Jun Guo, Yupeng He, Debra Ferguson, Sujatha Jagadeeswaran, Donald J. Osterling, Wenqing Gao, Julie K. Spence, Marina Pliushchev, Ramzi F. Sweis, Fritz G. Buchanan, Michael R. Michaelides, Alexander R. Shoemaker, Chris Tse, Gary G. Chiang

**Affiliations:** Discovery Research, AbbVie Inc., 1 North Waukegan Road, North Chicago, IL 60064 United States of America; Peking University Health Science Center, CHINA

## Abstract

Histone methyltransferases are epigenetic regulators that modify key lysine and arginine residues on histones and are believed to play an important role in cancer development and maintenance. These epigenetic modifications are potentially reversible and as a result this class of enzymes has drawn great interest as potential therapeutic targets of small molecule inhibitors. Previous studies have suggested that the histone lysine methyltransferase G9a (EHMT2) is required to perpetuate malignant phenotypes through multiple mechanisms in a variety of cancer types. To further elucidate the enzymatic role of G9a in cancer, we describe herein the biological activities of a novel peptide-competitive histone methyltransferase inhibitor, A-366, that selectively inhibits G9a and the closely related GLP (EHMT1), but not other histone methyltransferases. A-366 has significantly less cytotoxic effects on the growth of tumor cell lines compared to other known G9a/GLP small molecule inhibitors despite equivalent cellular activity on methylation of H3K9me2. Additionally, the selectivity profile of A-366 has aided in the discovery of a potentially important role for G9a/GLP in maintenance of leukemia. Treatment of various leukemia cell lines *in vitro* resulted in marked differentiation and morphological changes of these tumor cell lines. Furthermore, treatment of a flank xenograft leukemia model with A-366 resulted in growth inhibition *in vivo* consistent with the profile of H3K9me2 reduction observed. In summary, A-366 is a novel and highly selective inhibitor of G9a/GLP that has enabled the discovery of a role for G9a/GLP enzymatic activity in the growth and differentiation status of leukemia cells.

## Introduction

Epigenetic alterations to the genome take place by covalent modifications to the DNA or histones and result in changes in gene expression that do not arise from changes in the underlying DNA sequence. Histone post-transcriptional modifications can occur at specific amino acids with a diverse set of chemical modifications including acetylation, methylation, phosphorylation, SUMOylation and ubiquitination [1]. Among these modifications, histone methylation is induced at lysine or arginine residues by histone methyltransferases (HMTs), which catalyze mono-, di-, and/or trimethylation on lysine residues and mono- or di-methylation methionine (SAM) as the cofactor/methyl donor [2]. Generally, histone lysine methylation is associated with epigenetic regulation of the structure of chromatin and gene expression [3, 4]. HMTs have recently generated increased interest as potential targets of therapeutic value in human disease, especially since these epigenetic modifications are reversible [5, 6]. For example, a number of small molecule inhibitors generated against HMTs including EZH2 [7] and DOT1L [8] have shown potential benefit in preclinical models of diffuse large B-cell lymphoma and mixed lineage leukemia, respectively. As a result, targeting HMT activity has been the subject of heavy interest within the drug discovery field and several clinical trials have been initiated [1].

G9a (also known as EHMT2 or KMT1C) and the closely related GLP (G9a-like protein, also known as EHMT1 or KMT1D) are HMTs that share 80% sequence identity in their catalytic domains and are believed to form homo- and hetero-dimers [9]. G9a and GLP are amongst a set of HMTs known to catalyze the mono- and di-methylation of lysine 9 on histone 3 (H3K9me1/2) [9, 10]. H3K9-me1/2 is a highly abundant chromatin modification that is enriched at inactive gene loci [11] and CpG islands [12]. Both G9a and GLP have also been reported to di-methylate the tumor suppressor p53 at lysine 373, resulting in inactivation of p53’s transcriptional activity [13]. Several reports have highlighted the potential link of G9a to a variety of cancers. G9a is ubiquitously expressed in somatic cells but has been reported to be upregulated in a number of cancer types including leukemias [13], prostate cancer [14], hepatocellular carcinoma [15] and lung cancer [16]. Additionally, elevated expression of G9a in aggressive lung cancer correlates with poorer prognosis and knockdown of G9a in highly invasive lung cancer cells suppressed metastasis in a mouse tumor model [17].

The first disclosed selective small molecule inhibitor of G9a was BIX01294, which was shown to decrease global H3K9me2 in mouse embryonic stem (ES) cells and fibroblasts [18]. More recent G9a/GLP inhibitors are derivatives of this original quinazoline-based inhibitor and are frequently used to ascertain the cellular function of G9a catalytic activity. Compared to BIX01294, these next generation inhibitors, such as UNC0638, demonstrate improved cellular activity and separation between H3K9me2 reduction and toxicity between cell lines [19]. To further interrogate the role of G9a enzymatic activity in oncogenic processes we have generated A-366, a chemically distinct small molecule inhibitor of G9a [20]. A-366 is a peptide-competitive inhibitor of G9a/GLP with an enzymatic IC_50_ of ~3 nM and >100-fold selectivity over other methyltransferases and other non-epigenetic targets [20]. We find that A-366 has significantly less cytotoxic effects compared to other known G9a/GLP small molecule inhibitors despite roughly equivalent cellular potency in inhibiting the methylation of H3K9me2. The novel chemical scaffold has also allowed us to interrogate the contribution of G9a to the maintenance of leukemia cells. Our data indicate that A-366 is an improved small-molecule probe of G9a/GLP activity that will benefit the assessment of these epigenetic modifiers in cancer and potentially other indications.

## Materials and Methods

### Cell culture and cellular proliferation analysis

All tumor cell lines were obtained from American Type Culture Collection (ATCC). All tumor cell lines were grown according to manufacturer’s conditions in the recommended growth media in the presence of 10% fetal bovine serum (HyClone). Cell lines were tested and authenticated by short tandem repeat analysis using the GenePrint 10 System (Promega) and capillary electrophoresis (ABI Prism 3130). Primary human peripheral blood mononuclear cells (PBMCs) from two donors were obtained through BioreclamationIVT, pooled together and grown in AIM-V medium (Invitrogen). Viable cell numbers were measured using CellTiter-Glo reagent (Promega) as an endpoint assay. Inhibition was quantified as the ratio of treated cells compared to non-treated controls relative to cell free wells as 100% inhibition.

### Antibodies, western blotting and in-cell westerns

Antibodies used included anti-H3K9me2 (AbCam, ab1220), anti-total Histone H3 (Cell Signaling Technology, #4499), anti-G9a (Cell Signaling Technology, #3306), anti-H3K4me2 (Cell Signaling Technology, #9725), anti-H3K4me3 (Cell Signaling Technology, #9751), anti-H3K9me3 (Cell Signaling Technology, #9754), anti-H4K20me1 (Cell Signaling Technology, #9724), anti-H4K20me3 (Cell Signaling Technology, #5737), anti-H3K36me2 (Cell Signaling Technology, #2901), and anti-H3K27me3(Cell Signaling Technology, #9733). Western blots were performed as previously described [21]. For In-Cell westerns, PC3 cells were plated at 5,000 cells per well and allowed to adhere overnight. The next day, indicated concentrations of small molecule inhibitors or DMSO were added. Cells were then incubated at 37°C (5% CO_2_) for 72 hours. Cells were then fixed with 3% (w/v) formaldehyde/PBS for 15 minutes. After five washes with 0.1% (v/v) Triton X-100 in PBS, cells were blocked for 1 hour with 1% (w/v) BSA in PBS, then incubated with primary H3K9me2 mouse antibody at 1:500 and total Histone H3 rabbit antibody at 1:1000 in 0.3% BSA and PBS for 4 hours. Secondary fluorescence-conjugated antibodies (LiCor Biosciences) were added for 1 hour. After five washes with 0.1% Tween-20 in PBS, the plates were read on an Odyssey (LiCor Biosciences) scanner at 800 nm (H3K9me2 signal) and 700 nm (total histone H3 signal). Fluorescence intensity was quantified using the Licor Odyssey imaging suite software.

### Analysis of cell cycle and CD11b by flow cytometry

MV4;11 or HL-60 cells were incubated with the indicated concentrations of A-366 for the indicated number of days. Cells were collected and then washed with cold PBS before staining with 0.5ml PBS containing 50 μg/ml propidium iodide (PI), 0.1mg/ml RNase A, 0.1% BSA, and 0.1% Triton-X100 for 20 minutes. Cell cycle distribution was analyzed using a BD LSR-II flow cytometer (BD Biosciences). FACS analysis of CD11b expression was performed on duplicate cell samples using PE-conjugated human CD11b-specific mouse monoclonal antibody at a 1:5 dilution (BD Pharmingen) on a BD LSR11 FACS machine.

### Clonogenic assays

Live cells were washed and seeded at 500 cells per well into six-well plates in drug-free medium. 24 hours later, compounds were diluted in the appropriate cellular media and added to the cells. Cells were cultured at 37°C for 7–10 days. Cells were then fixed and stained with 0.2% crystal violet to visualize and count colonies using a GelCount colony counter (Oxford Optronix).

### Wright-Giemsa staining

MV4;11 cells were treated with DMSO or 5 μM A-366 for 10 days. Following incubation, treated cells were stained using a Wright-Giemsa kit (Polysciences, Inc.).

### H3K9me2 cellular AlphaLISA

Cells were treated for 48 hours with the indicated concentrations of compound and assessed for viability using CellTiter-Glo reagent (Promega). H3K9me2 levels were measured utilizing the H3K9me2 AlphaLISA assay system (PerkinElmer) and normalized to cell viability. All values were then normalized to non-treated cells as 0% inhibition of H3K9me2.

### *In vivo* studies

All animal studies were conducted in accordance with the guidelines established by internal Institutional Animal Care and Use Committees at AbbVie, Inc. (North Chicago, IL). Tumor cells were mixed with 50% Matrigel (BD Biosciences, San Jose, CA) and 5 x 10^6^ cells per mouse were injected subcutaneously into the flank of 6–8 week old SCID-beige female mice (Charles River Laboratories, Wilmington, MA). The tumors were allowed to grow to approximately 200 mm^3^, at which time mice were allocated by tumor volume into study groups (n = 10 mice/ group) so that the mean tumor volumes of the groups were statistically similar. Mice were then entered into the dosing phase of the study (described below) and tumor volumes were recorded 2 times per week. Tumor volumes were estimated by the formula V = (L x W^2^)/ 2, where V is the volume (mm^3^), L is the tumor length (mm) and W is the tumor width (mm), measured at right angles using a digital caliper. The effect of a treatment on tumor growth inhibition was determined as %TGI = mean tumor volume of control group—mean tumor volume of treatment group/ tumor volume of treatment control group x 100. A-366 was formulated for delivery by osmotic mini-pump in 98% PEG-400 and 2% Tween-80.

## Results

### A-366 is a cell-active G9a/GLP small molecule inhibitor

**Fig 1:**
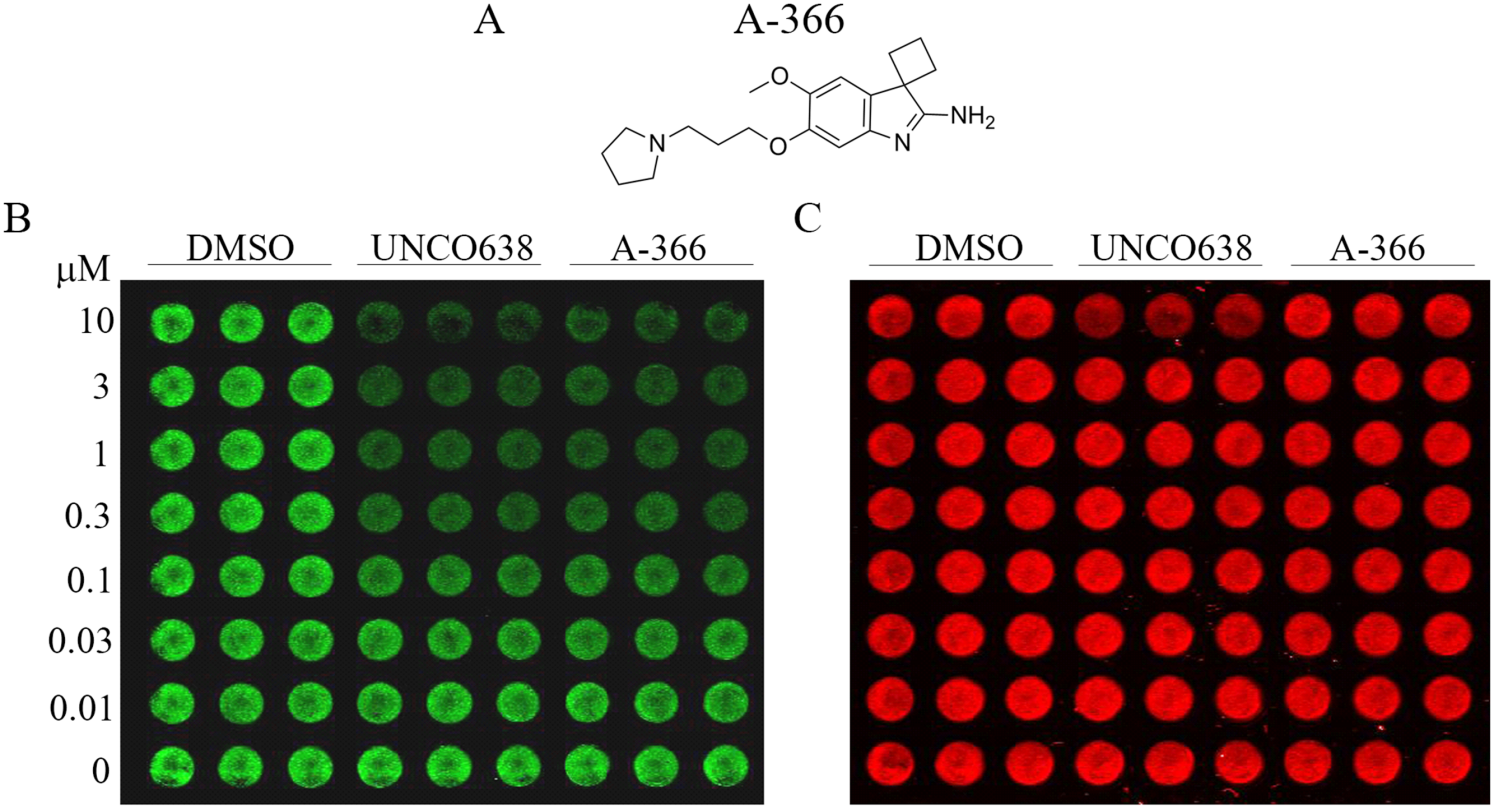
A-366 has cellular activity comparable to known G9a/GLP inhibitors. (A) The chemical structure of A-366. (B) PC-3 prostate adenocarcinoma cells were incubated in triplicate with DMSO or the indicated concentrations of A-366 or UNC0638 for 72 hours. H3K9me2 levels were assessed by In-Cell Western assay. (C) In-Cell Western assay for total histone H3 levels from the same plate as shown in (B).

In order to identify chemically distinct small molecule inhibitors of G9a, we performed a high-throughput screen of a chemical diversity subset of our internal compound collection. A singleton hit (spiro[cyclobutane-1,3’-indol]-2’amine) ultimately led to the generation of A-366 (Fig 1A, [20]). A-366 is a peptide-competitive inhibitor of G9a/GLP with an *in vitro* enzymatic IC_50_ of ~3 nM and >100-fold selectivity over other methyltransferases and other non-epigenetic targets [20]. The potency and selectivity of A-366 made it an ideal candidate to probe the cellular activities of G9a/GLP. To assess the relative cellular activity of A-366, we treated the human prostate adenocarcinoma cell line PC-3 with A-366 or the control compound UNC0638 before performing in-cell western blotting to measure histone H3 lysine 9 methylation. A-366 reduced the total levels of H3K9me2 in a time and concentration dependent manner with a cellular EC_50_ of ~300 nM (Fig 1B), similar to UNC0638. Full reduction of the H3K9me2 signal required at least 3 days of incubation, which is consistent with other inhibitors with an epigenetic mechanism of action (data not shown, [7, 19]). A-366 was also specific for dimethylation of H3 lysine 9 when compared to a panel of eight additional histone methyl modification antibodies (S1 Fig) as suggested by previous biochemical data [20]. In addition, treatment of cells with 10 μM UNC0638 showed reduced amounts of total H3 staining, which is suggestive of cytotoxicity, although this was not observed with A-366 treatment (Fig 1C). These results suggest that A-366 is a potent and cell-active inhibitor of G9a/GLP that could be used for further investigation of the biological functions of G9a/GLP.

### A-366 does not impact proliferation in MCF-7 cells

**Fig 2:**
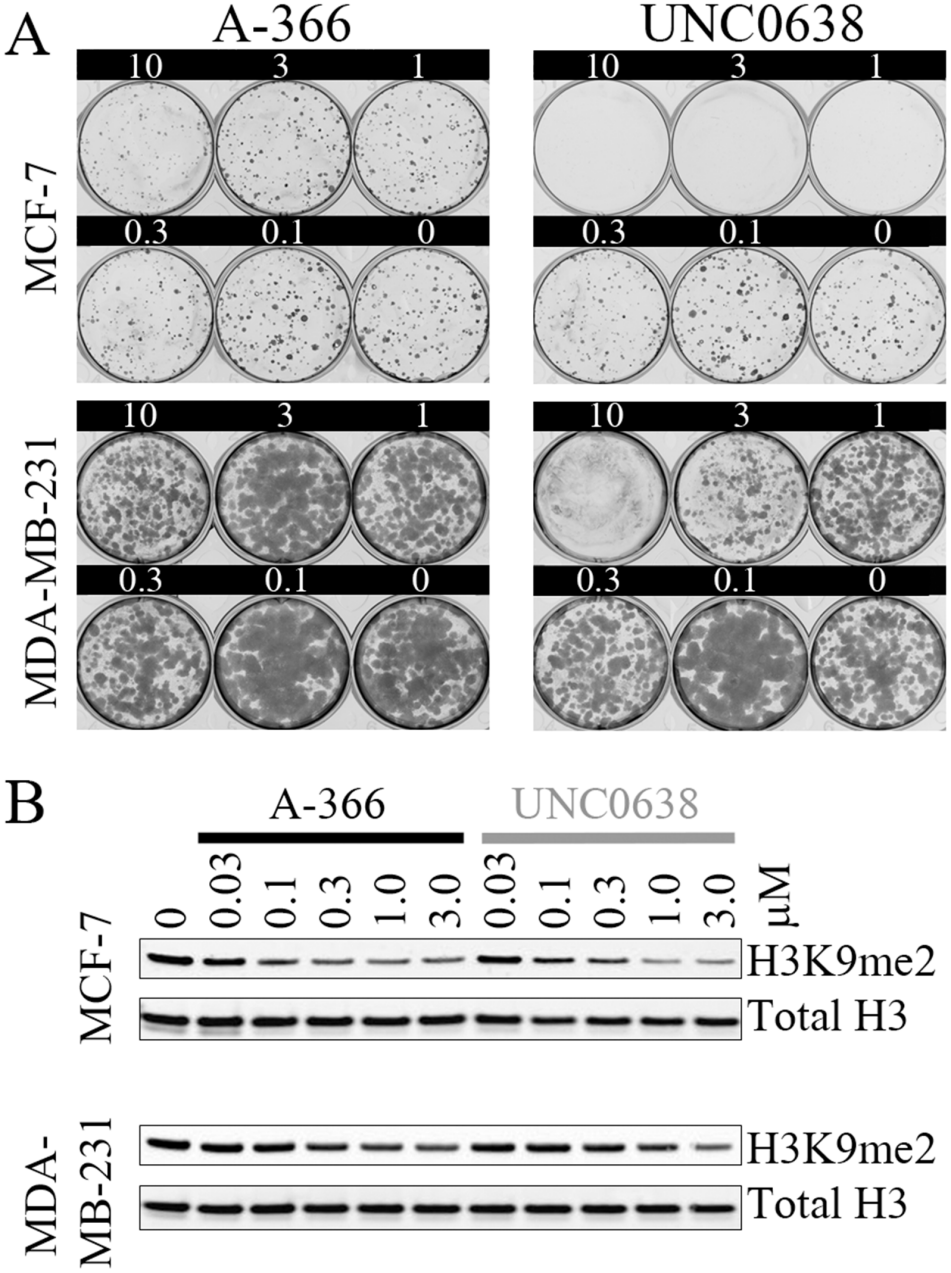
A-366 does not impact the proliferation of MCF-7 cells despite inhibition of H3K9me2. (A) MCF-7 and MDA-MB-231 breast cancer cell lines were treated with the indicated concentrations of A-366 or UNC0638 for 14 days before assessing colony formation. (B) MCF-7 and MDA-MB-231 cells were treated with the indicated concentrations of A-366 or UNC0638 for 3 days and subjected to western blot analysis of global H3K9me2 and total histone H3.

A previous report suggested that inhibition of G9a/GLP by RNA interference or continuous dosing with UNC0638 reduced the clonogenicity of MCF-7 but not MDA-MB-231 breast cancer cells [19]. We attempted to recapitulate this result with our novel G9a/GLP inhibitor, A-366. While the treatment of MCF-7 cells with UNC0638 reproduced the published results, A-366 had no impact on cellular clonogenicity (Fig 2A). Neither compound affected MDA-MBA-231 cells in this assay format, with the exception of the highest concentration tested for UNC0638 (10 μM). Notably, even 14 days of treatment with 10 μM A-366 had no impact on the growth of either of these cell lines despite being well above the cellular EC_50_ shown in Fig 1B. To test whether inadequate target inhibition might account for these observations, we assessed these compounds’ impact on global H3K9me2 levels in both breast cancer cell lines. However, A-366 and UNC0638 exhibited nearly identical cellular potency in reducing global H3K9me2 signal (Fig 2B). The treatment of MCF-7 cells consistently resulted in more H3K9me2 reduction than in the MDA-MB-231 cells, suggesting that the MDA-MB-231 cell line may have more contribution from additional H3K9 methyltransferases or that the turnover of H3K9me2 by histone demethylases may be more rapid in MCF-7 cells.

### A-366 has disparate effects on cell viability compared to UNC0638 despite equivalent inhibition of global H3K9me2

**Fig 3:**
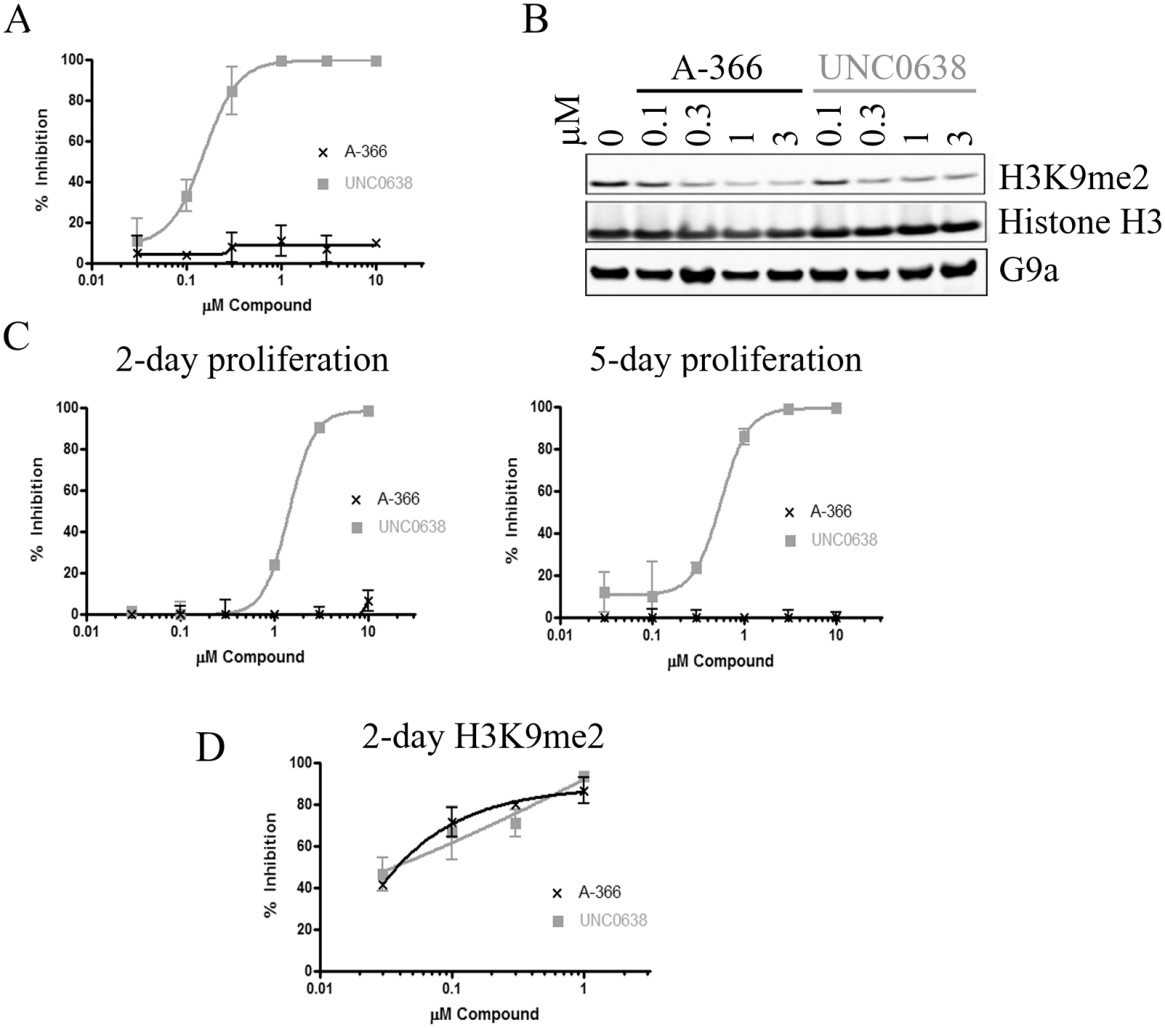
A-366 inhibits H3K9me2 similar to UNC0638, but does not inhibit cell growth of MOLT-16 and HT-1080 cells. (A) The T-cell ALL cell line MOLT-16 was treated for 5 days with A-366 or UNC0638 before assessing their proliferation. (B) MOLT-16 cells were treated for 3 days with compounds and lysed. Lysates were normalized and western blot analysis was performed for H3K9me2, total H3 and G9a. (C) HT-1080 fibrosarcoma cells were treated for 2 or 5 days with A-366 or UNC0638 before assessing their proliferation. (D) H3K9me2 AlphaLISA was performed on HT-1080 cells treated for 2 days with compounds and % inhibition of H3K9me2 was calculated compared to untreated control cells.

The discrepancy in cell proliferation EC_50_s for A-366 and UNC0638 despite equivalent EC_50_s for H3K9me2 inhibition suggested that UNC0638 may have off-target effects. To test this hypothesis, we treated additional cell lines with A-366 compared to UNC0638. In the T-cell acute lymphoblastic leukemia (ALL) cell line MOLT-16, UNC0638 inhibited cell proliferation at concentrations consistent with its H3K9me2 EC_50_; however, A-366 had no impact on the proliferation of this cell line even though it inhibited H3K9me2 as potently as UNC0638 (Fig 3A and 3B). We further assessed the effects of these compounds in the human fibrosarcoma cell line HT-1080. UNC0638 exhibited anti-proliferative effects after only 2 days of treatment (1.4 μM EC_50_, Fig 3C). Treatment for an additional 3 days resulted in a reduction of the proliferation EC_50_ to 0.5 μM (Fig 3C), in line with the observed H3K9me2 cellular EC_50_ of 0.3 μM. However, A-366 had no impact on HT-1080 proliferation up to 10 μM. The cellular inhibition of G9a/GLP is also not consistent with these results as these two compounds have nearly identical impact on global H3K9me2 levels in this cell line at the relevant concentrations of interest (Fig 3D). We also performed an unbiased screen of an additional 38 cell lines from numerous cancer types to assess the impact of A-366 (S1 Table). We found that A-366 had no overt anti-proliferative effects in any cell line tested up to 10 μM, often in contradiction to the quinazoline-based inhibitor UNC0638. Finally, we tested A-366 in human PBMCs and found that A-366 also had no impact on viability on these non-transformed cells (S2 Fig).

### Long term treatment of leukemia cell lines with A-366 results in differentiation and inhibition of growth

**Fig 4:**
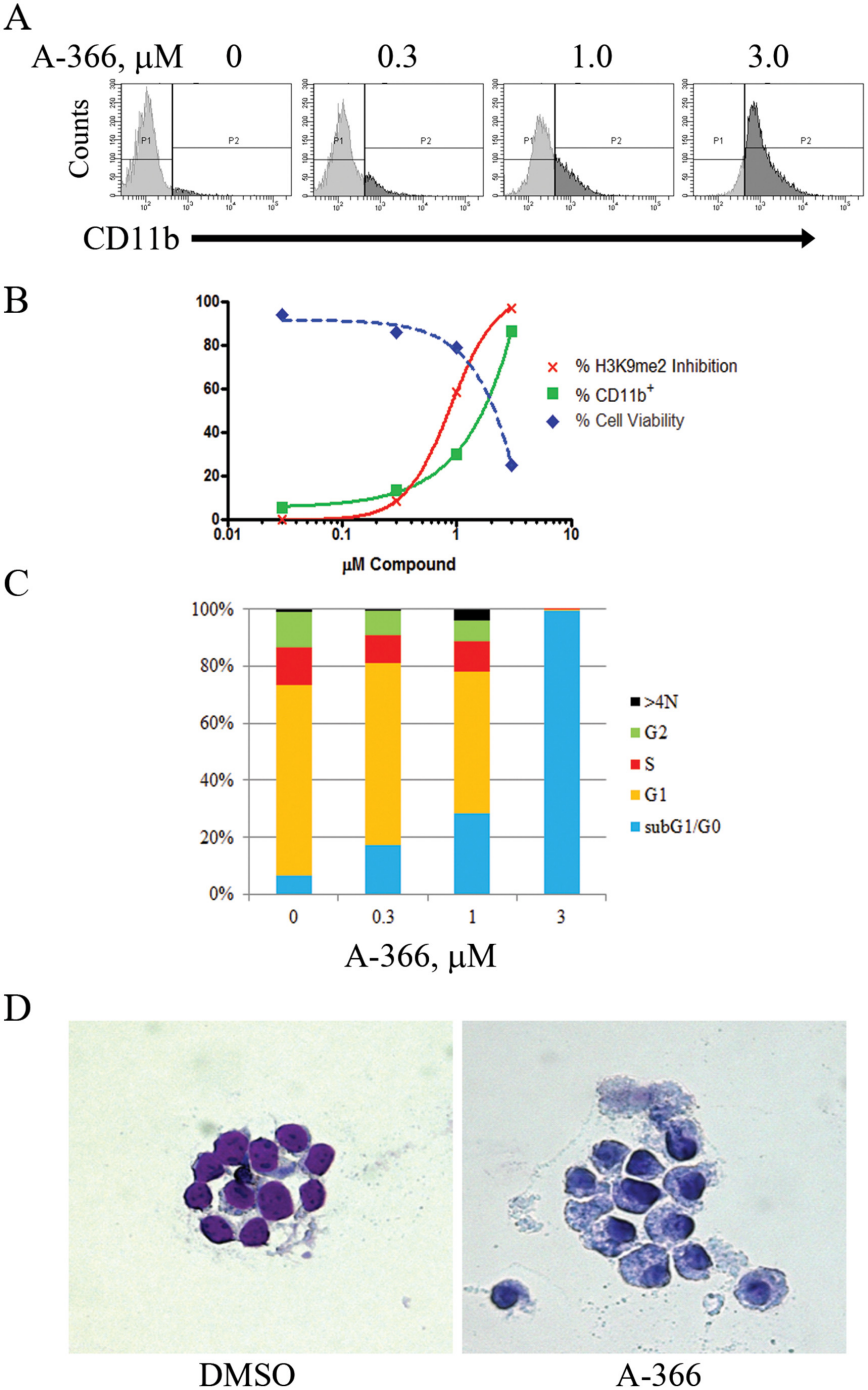
A-366 induces differentiation and affects viability in MV4;11 cells. (A) MV4;11 cells were incubated with A-366 for 14 days. Cells were fixed, stained with an anti-CD11b antibody and analyzed by flow cytometry. (B) MV4;11 cells from (A) additionally assayed for cellular proliferation (Cell Titer-Glo) and viability (trypan blue exclusion) following A-366 treatment for 14 days. (C) Cell cycle DNA content analysis was performed by propidium iodide staining in MV4;11 cells treated for 14 days with A-366. (D) Wright-Giemsa staining of MV4;11 cells treated with DMSO or 5 μM A-366 for 10 days.

One of the characteristic features of leukemia cells is a blockade of differentiation at a distinct stage of blood cell maturation [22]. As a result, differentiation therapy has been tested and validated clinically in several oncology indications. For example, two established clinical regimens for differentiation therapy, the treatment of acute promyelocytic leukemia with all-trans retinoic acid (ATRA) [23] and azacitidine treatment of myelodysplastic syndrome [24], provide proof-of-principle for this approach. Recently, G9a has been implicated in mediating T helper cell differentiation and function by studies of tissue-specific, G9a conditional knockout mice [25]. We hypothesized that inhibiting G9a function may likewise translate to the inhibition of leukemia cells by differentiation. As a first test of this hypothesis, we treated the acute myelocytic leukemia (AML) cell line MV4;11 with A-366. Long-term treatments resulted in dose-dependent differentiation of this tumor cell line, as observed by an increase in the differentiation marker CD11b (Fig 4A). A-366 treatment also resulted in inhibited proliferation and a decrease in viability corresponding to the dose response observed for CD11b staining (Fig 4B). DNA content analysis was also performed to assess a potential mechanism of action for the reduced viability observed in MV4;11 cells treated long-term with either A-366. We found that MV4;11 cells underwent a dose-dependent transition towards sub G_1_/G_0_ content consistent with the dose response observed for CD11b differentiation of this cell line (Fig 4C). Of note, we observed that shorter treatments (3 and 7 days) with A-366 produced similar but less dramatic effects, consistent with the delayed kinetics of an epigenetic mechanism of action (data not shown). Finally, we observed morphological changes following A-366 treatment, such as increased cytoplasm:nucleus ratio and nuclear lobulation, indicative of differentiation (Fig 4D).

We also treated the acute promyelocytic leukemia (PML) cell line HL-60 with A-366. As opposed to the differentiation and ultimate decrease in viability seen with A-366-treated MV4;11 cells, short or long-term treatment (4 or 14 days, respectively) of HL-60 cells resulted in a dose-dependent differentiation and a corresponding decrease in proliferation (S3A and S3B Fig). However, DNA content analysis of A-366-treated HL-60 cells showed an accumulation of cells in G_1_ consistent with cytostasis (S3C Fig).

### A-366 treatment of MV4;11 xenografts elicits growth inhibition *in vivo*

**Fig 5:**
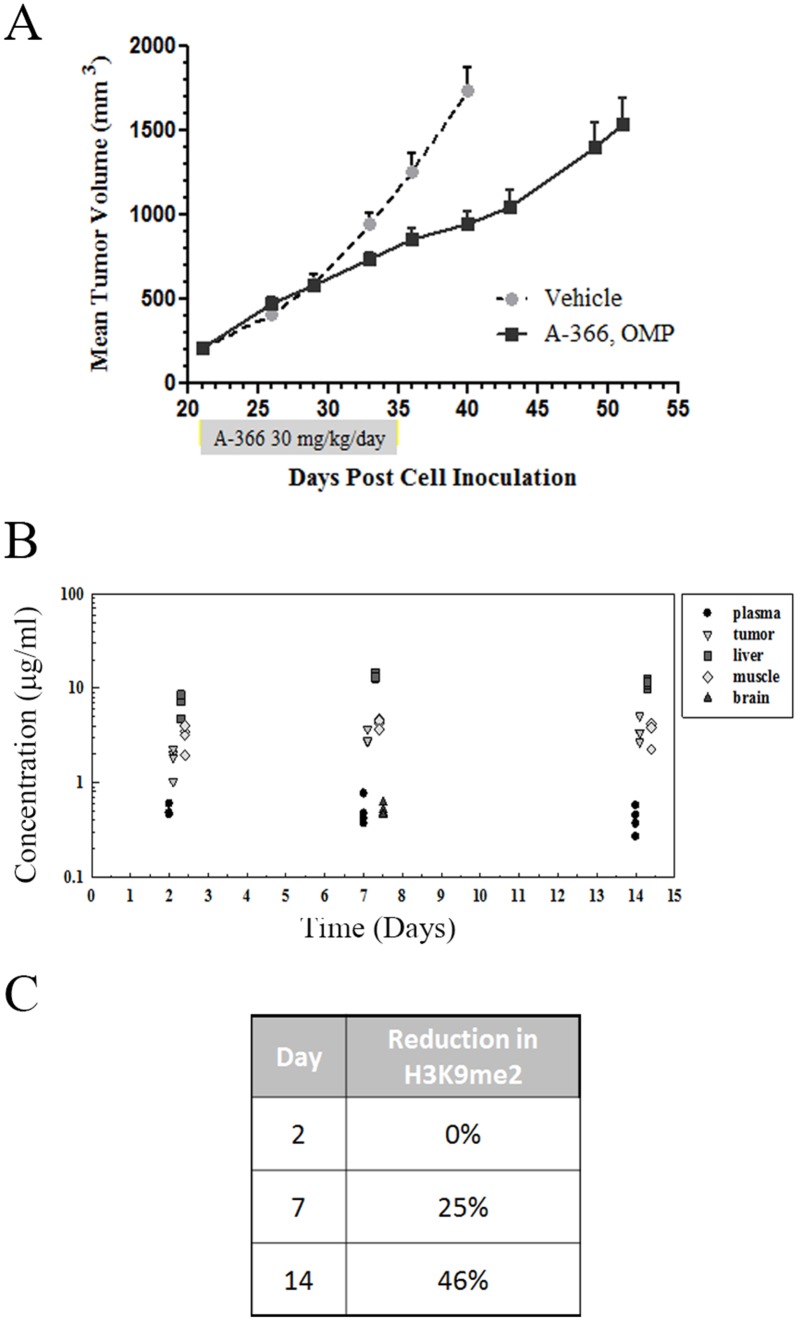
*In vivo* efficacy study of A-366 in MV4;11 flank xenografts. (A) MV4;11 cells were implanted subcutaneously in SCID/bg mice and allowed to establish tumors of ~200 mm^3^. A-366 was administered to tumor-bearing mice at 30 mg/kg/day by osmotic mini-pump for 14 days. Tumors were measured at the indicated time points and tumor growth was plotted as a function of time. (B) In a parallel PK/PD arm carried out in MV4;11 tumor-bearing animals, plasma and tissues were collected at the indicated time points and analyzed for A-366 levels. (C) A-366-treated tumors from the indicated time points were harvested and analyzed by AlphaLISA for reductions in H3K9me2 relative to vehicle.

To assess the potential role that G9a/GLP inhibition may have in an *in vivo* setting we tested A-366 in a AML flank xenograft model using MV4;11 cells. However, initial MTD studies with A-366 dosed intraperitoneally (IP) showed toxicity in the animals at 10 mg/kg, which would limit exposures to a level not predicted to be efficacious for inhibiting tumor growth. To achieve a projected efficacious exposure while mitigating the presumed C_max_-driven toxicity, osmotic mini-pump (OMP) dosing was utilized. Mice treated with either vehicle control or A-366 administered via osmotic mini-pump at 30 mg/kg/day for two weeks showed no overt toxicity. We observed a modest 45% tumor growth inhibition resulting from A-366 treatment in this model (Fig 5A). During this *in vivo* efficacy trial, a separate arm of tumor-bearing size matched cohorts was sacrificed after 2, 7 and 14 days of A-366 treatment. Plasma and tissue specimens were collected from these treated tumor-bearing animals to assess the PK/PD relationship to the efficacy arms. We found that A-366 had significant accumulation in the tumors and other tissues tested (Fig 5B). Finally, the levels of global H3K9me2 were assessed in the MV4;11 tumors from mice in the PK/PD arm of the study and we found a slow but steady decrease in total H3K9me2 levels (Fig 5C). The combination of modest *in vivo* efficacy in MV4;11 xenografts aligns well with the slow kinetics of H3K9me2 inhibition despite the significant accumulation of A-366 observed in these tumors.

## Discussion

Interest in small molecule inhibitors of HMTs has surged recently as these enzymes have emerged as targets of potential therapeutic value. Based on literature reports and our own internal data, we sought to generate chemically distinct inhibitors of G9a to validate its role in oncogenic processes. A-366 has sub-μM cellular potency on H3K9me2 coupled with minimal effects on cellular proliferation at concentrations up to 10 μM which is an improvement upon previously published quinazoline-based G9a/GLP small molecule inhibitors. The unique combination of potency and selectivity observed with A-366 enabled testing of the hypothesis that G9a plays a role in leukemia cell differentiation. This was accomplished through long-term cell culture models required to reverse the epigenetic mechanisms responsible for suppression of differentiation of these tumors. During the preparation of this manuscript, Lehnertz, *et al*. described a conditional knockout mouse model with G9a ablated in the hematopoietic lineage that defined a role for G9a in regulation of HoxA9-dependent transcription in AML. Importantly, they demonstrated that deletion of G9a in HOXA9/Meis1 transformed murine HSPCs delayed the progression to end stage AML in transplanted mice [26]. Our data corroborate and extend their findings, demonstrating that pharmacological modulation of G9a can inhibit growth of an AML xenograft model *in vivo*. It should be noted that the modest results observed with the MV4;11 flank xenograft model may point to the potential difficulties associated with treating rapidly-growing, established tumors with this type of epigenetic therapy. Therefore, perhaps a better approach would be to combine standard of care cytotoxic agents with epigenetic therapies such as A-366 in order to achieve a more durable response clinically. Nevertheless, we observed efficacy with A-366 as a single agent and these effects can be directly correlated to the levels of H3K9me2 inhibition observed especially considering the reduced off-target effects of this improved small molecule inhibitor.

Numerous studies have been published suggesting additional roles for G9a/GLP in various biological indications beyond oncology including mental retardation [27], cocaine addiction [28], maintenance of HIV-1 latency [29] and autophagy [30]. The generation and disclosure of A-366 will be of great interest in further validation studies for these hypotheses as it will allow for more specific dissection of G9a/GLP function than previously disclosed small molecule G9a/GLP inhibitors. During our characterization of A-366, we tested published quinazoline-based inhibitors of G9a/GLP [31] and numerous additional analogs of the quinazoline series produced in-house and found that many reported biological activities of track with the quinazoline core of the molecule versus the G9a/GLP activity of these compounds (data not shown). While UNC0638 does notably possess potent inhibition of G9a/GLP, our data raise a cautionary note in ascribing the biological phenotypes arising from UNC0638 treatment to G9a/GLP function. In summary, A-366 is a novel small molecule inhibitor of the HMTs G9a and GLP that enabled pharmacologic validation of the hypothesis that G9a plays a key role in the epigenetics of leukemia maintenance and differentiation and that treatment of these tumor types with selective, bioactive inhibitors of G9a may be clinically relevant.

## Supporting Information

S1 Fig PC-3 prostate adenocarcinoma cells were incubated in with DMSO, 0.3 or 3 μM of A-366 for 72 hours. Methyl mark levels were assessed by western blot analyses.
(TIF)

S2 Fig Human PBMCs were treated for 48 hours with compounds and an endpoint assay was performed with Cell-Titer Glo to assess viability. (TIF)

S3 Fig (A) HL-60 cells were incubated with A-366 for 4 days. Cells were fixed, stained with an anti-CD11b antibody and analyzed by flow cytometry. (B) HL-60 cells from (A) were assessed for cellular proliferation (Cell Titer-Glo) and viability (trypan blue exclusion) following 4 days of treatment with A-366. (C) Cell cycle DNA content analysis was performed by propidium iodide staining in HL-60 cells treated for 4 days with A-366.
(TIF)

S1 Table Table of EC50 values for A-366 and UNC0638 in a panel of tumor cell lines as determined by Cell Titer-Glo assay after incubation for the times listed allowing for sufficient proliferation. (PDF)
