# Supplementary figures and images for: The Histone Methyltransferase Inhibitor A-366 Uncovers a Role for G9a/GLP in the Epigenetics of Leukemia
Source: PLoS One. 2015 Jul 6;10(7):e0131716. doi: 10.1371/journal.pone.0131716 (PMC4492996; doi:10.1371/journal.pone.0131716)

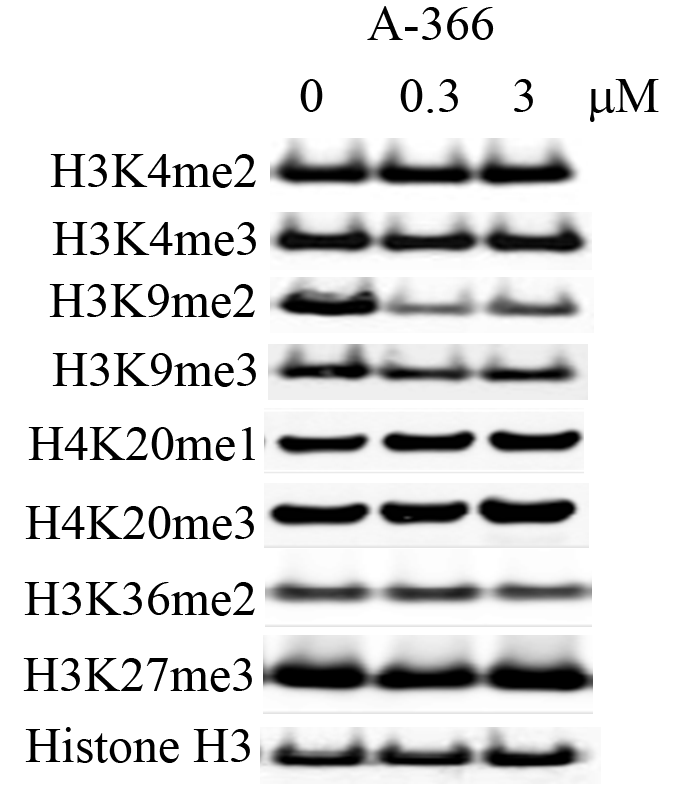

Supplement: S1 Fig — Methyl mark levels were assessed by western blot analyses. (TIF) [file pone.0131716.s001.tif]

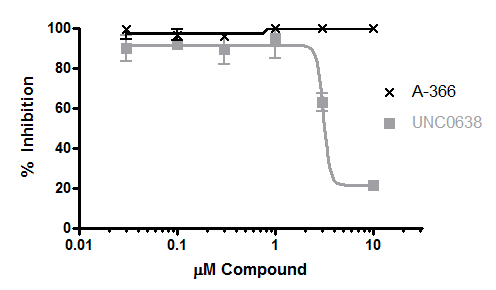

Supplement: S2 Fig — (TIF) [file pone.0131716.s002.tif]

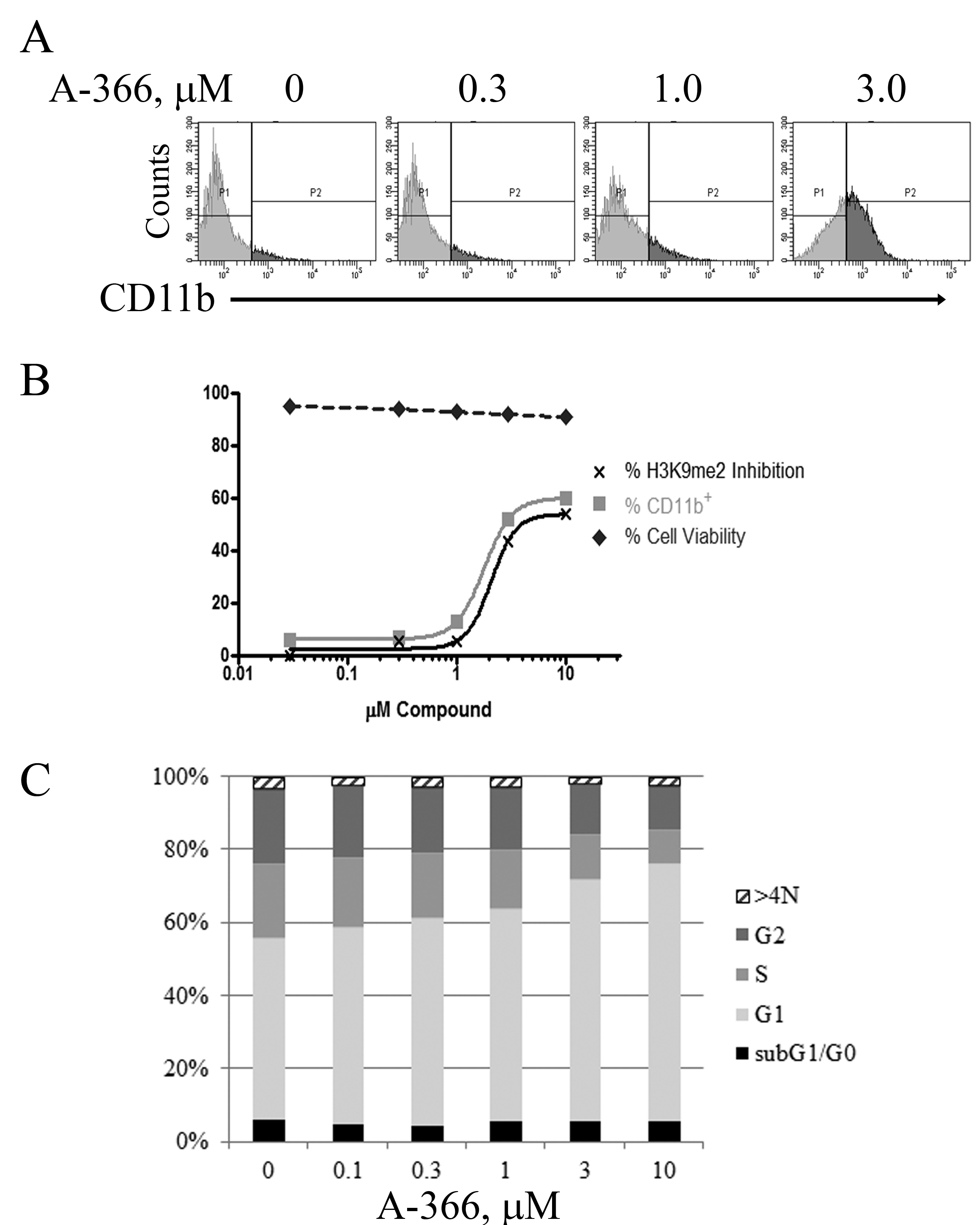

Supplement: S3 Fig — (A) HL-60 cells were incubated with A-366 for 4 days. Cells were fixed, stained with an anti-CD11b antibody and analyzed by flow cytometry. (B) HL-60 cells from (A) were assessed for cellular proliferation (Cell Titer-Glo) and viability (trypan blue exclusion) following 4 days of treatment with A-366. (C) Cell cycle DNA content analysis was performed by propidium iodide staining in HL-60 cells treated for 4 days with A-366. (TIF) [file pone.0131716.s003.tif]
