# Supplementary material for: The Histone Methyltransferase Inhibitor A-366 Uncovers a Role for G9a/GLP in the Epigenetics of Leukemia
Source: PLoS One. 2015 Jul 6;10(7):e0131716. doi: 10.1371/journal.pone.0131716 (PMC4492996; doi:10.1371/journal.pone.0131716)
Supplement: S1 Table — (PDF) [file pone.0131716.s004.pdf]

S1 Table.

	5-day proliferation	
**Cell line**	**A-366**	**UNC0638**
	**EC_{50} μM**	**EC_{50} μM**
MOLT-16	>10	0.2
THP-1	>10	4.9
RS4;11	>10	3.1
RPMI8226	>10	5.1
OPM2	>10	5.6
L-363	>10	3.9
KMS12	>10	4.4
KMS11	>10	4
Kasumi-1	>10	1.1
HL-60	>10	6.5
H929	>10	4.6
MV4;11	>10	1.7
K562	>10	5.3
22RV1	>10	0.2
HT-1080	>10	0.5
Calu6	>10	0.5
DU145	>10	0.7
LN229	>10	0.5
LNCap	>10	0.3
SKBR3	>10	0.6
H1975	>10	>10
H2009	>10	3.9
Calu-1	>10	1.3
Calu-6	>10	1.5
H358	>10	4.3
H441	>10	8.7
H522	>10	1.5
H661	>10	4.9
	**7-day proliferation**	
**Cell line**	**A-366**	**UNC0638**
	**EC50 μM**	**EC50 μM**
MHH-65-1	>10	1.9
CAPO-ES1	>10	1.2
PA-1	>10	0.2
SK-N-MC	>10	0.2
G-401	>10	0.4
ZR7350	>10	1.2
U2OS	>10	0.5
DaoY	>10	0.1
PH-1	>10	4.4
TC-71	>10	0.6

	12-day proliferation	
**Cell line**	**A-366**	**UNC0638**
	**EC_{50} μM**	**EC_{50} μM**
IMR-32	>10	0.1
A-201	>10	0.6
